# An Important Question Answered

**Published:** 1885-03

**Authors:** Henry C. Suess

**Affiliations:** Burlington, Iowa


### AN IMPORTANT QUESTION ANSWERED.

HENRY C. SUESS, M. D., BURLINGTON, IOWA.

[Read before the Hahnemann Medical Association of Iowa, May 28th, 1884.]

In religion, much depends on what we believe; in medicine, more on what we know. This truth applies to us as homœopathists especially.

The question very naturally presents itself: What must we know in order to become successful practitioners of medicine?

I shall endeavor to answer this question under three heads, dwelling at some length on the last as being the most important one.

I. Every physician who subscribes to the homœopathic law of cure should possess a good education. In this respect Homœopathy need not be ashamed of her sons. Much of the progress she has made is in a great measure due to the excellent education of many of them. On the other hand, we are sorry to say that her onward march has been much impeded by the illiteracy of many others who claim recognition as homœopathists.

The action of our medical organizations here and there, but more that of some of our colleges, is very commendable and worthy of emulation, in that they admit to the study of medicine only such ladies and gentlemen that have given satisfactory evidence of possessing a good general education. It is very desirable that this movement become general all along the line of our colleges and societies.

Homœopathy should be first to elevate the standard. We live in a period marked by great discoveries and progress in the various branches of science. Ours is a time given to most critical investigation. Our most formidable opponents, the self-styled Regulars (?), exhibit great activity; their tactics have undergone great changes; they meet us less often in open hostility; they refrain from calling homœopathists knaves or fools. As an evidence of progress of our brethren of the Regular school (?) of medicine, this fact is especially worthy of mention.

Occasionally we even notice a desire on their part to consult with homœopathists. Undoubtedly, many of us will, ere long, have occasion to shake hands with them at the bedside. This will involve great responsibilities, and give us golden opportunities for the good of homœopathic medicine, the like of which we have never seen before.

When ignorant men become the champions of a cause, which in itself may be ever so true and right, they become its pall-bearers, conducting it out of sight. Through some such men reproach has occasionally been brought upon Homœopathy. I myself have seen the sign of these men read "*Homephatic Physician.*" Oh! how it vexed me! I felt ashamed for Homœopathy, that the man flung his ignorance to the breeze year in and out, into the face of allopathists of great learning and venerability.

But what shall we say in regard to the frequent display of ignorance on the part of correspondents in some of our medical journals? How long shall we bear it? The idea that men who, to put it mildly, do not even know the orthography of the terms used in their every way very ordinary writings, consider themselves foreordained to enlighten (?) others on subjects of which they know but little, if anything at all! Why such abortive productions are not consigned to the waste basket is a mystery to me. For one, I earnestly protest against this outrage perpetrated upon knowledge-seeking subscribers.

It seems to me there is but one way in which we can remedy this evil. Let us cancel our subscriptions to every such journal, giving our reasons for so doing, and ere long a marked improvement will be noticeable, and we shall no longer be held up to ridicule nor be forced to become laughing stock for our opponents through asinine would-be contributors.

II. Next we notice the necessity of proficiency in the various branches pertaining to the science and art of healing the sick. In the study of anatomy, physiology, pathology, etc., the physician lays the foundation for his therapeutic superstructure. It is of vital importance that this foundation be laid broad and deep.

He that has espoused the cause of ministering to suffering humanity in harmony with the divine law of cure, must make the structure of the tissues and organs of the human organism, physiologically and pathologically considered, also their functions as performed in health and modified by disease, a continuous study. But after all, while these reveal to the physician the nature of the disease, the course it naturally pursues, and the termination to be expected—in case therapeutic measures are not resorted to—the problem is not yet half solved. The great questions, What shall I do to save my patient? how shall I find the specific remedy for his complaint? still remain unanswered.

Pray, what does it benefit a patient that a man or a woman of great intelligence and brilliant attainments can give a correct diagnosis and prognosis of his case, if he or she is unable to bring relief? It is not sufficient that the physician has qualified himself to verify his expressed opinion at the *post-mortem*. The one great desideratum is, the gentle, speedy, and permanent cure of the sick.

Dr. Killmequick may be a thorough-going pathologist and real expert with the scalpel, but he lacks the principal prerequisite, the essential quality, that gains the favor of those in need of a physician, which, last but not least, is:

III. *A thorough knowledge of materia medica.*

This is really the one thing needful above all others. It is the magic wand by means of which the homœopathic physician finds ready entrance to the palaces of wealth and the temple of honor and fame. With it, all hindrances and obstacles are easily overcome. Against it, all opposition, individual and organized, however formidable in appearance, will ever prove puerile.

Without a liberal knowledge of the pathogeneses of the substances used as remedial agents, the physician is utterly unqualified to apply the law of the similars at the bedside, and unless he has this knowledge, or at least is earnestly endeavoring to obtain it, he should make way for others.

I fear very, very many of the rank and file of homœopathic physicians lack this great prerequisite to success. There are not a few who not even deemed it necessary to read the *Organon of Hahnemann*, while a greater number have never studied this important work. They may boast of possessing a copy. Very well. Let a diligent search be instituted and it will be found, all covered with dust, lying upon one of the upper shelves, in precisely the same condition in which it was when obtained and placed there. But more surprising than all this is the fact that many homœopathic physicians have not even seen the *Organon*. What good will such men do for Homœopathy? The works on materia medica remain undisturbed in the bookcase, whereas the stories of Captain Marryat and books of that ilk are always within ready reach, having greater attractions for them than the beautiful provings of our remedies found in the *Materia Medica Pura* and other works.

Now mark you the inevitable and unfortunate result that follows. The remedies which they administer in a haphazard way fail to produce curative effects, by degrees they lose what little confidence they ever had in Homœopathy, then they became disgusted with it, and finally they even deny the existence of laws that are well known and have been verified continually by hundreds, yea, by thousands, who have listened to the voice of the experience of the master and have accurately followed his precepts. They are like the storm-tossed vessel at sea, without mast and rudder, drifting hither and thither, falling, sooner or later, a prey to the wind and waves.

Such is the deplorable condition of the practitioners of medicine who neglect to make themselves acquainted with the drug provings and clinical verifications of Hahnemann, Hering, and others.

Show me a true homœopathic physician who does *not* study his *Materia Medica;* show him to me as being successful in the broadest sense of the word. Ah! you cannot do it. It is as impossible as it is to find a true Christian walking in the ways of truth without loving to study the Word of God daily.

Our day and generation seem to be given to gratification of self. Mental as well as physical labor is shunned. Many enter the profession with the erroneous idea that success must needs come to them if they but open an office, look wise as an owl, speak as a sage, wear the independence and air of a Gould or Vanderbilt, and—I came near adding—work like a beaver. But no. They are ease and pleasure-loving creatures, which, like butterflies, may be noticed here and there, but when you look for them they are nowhere to be found.

What does it benefit such physicians that Hahnemann and his colaborers, as also painstaking men since, have sunk the shaft and opened up a mine of wealth, leaving for us little else than to devise ways and means by which we may reach and sescure the treasure? This they propose to do in an easy, go-as-you-please way. They recline on the sofa, attend the opera, and, if unmarried, they flirt with the girls, when they should labor to obtain that knowledge which is power, of which the late lamented Carroll Dunham wrote: "That a person earnestly applying himself to the study of materia medica required seven years time to master it sufficiently to be enabled to prescribe intelligently and successfully." They are unable and unwilling to perform this difficult work, so they remain in comparative ignorance, possessing of Homœopathy only the name, which they value at zero, and are ready to drop at any time lest the finely organized and highly cultivated auditory nerves of some would-be Mogul of the dominant school be shocked. Homœopathy loses nothing by their departure, and the Regulars (?) gain but very little, since these pseudo-homœopathists never give away anything that is of any value to themselves.

While at one time these men beat an inglorious retreat, at another we hear of their negotiations with the enemy, endeavoring to make a compromise. Let us stand unitedly in the defense of the truth. Let us study our *Materia Medica* as if our eternal welfare depended on it. Let us not study pathology and kindred branches less, *but materia medica more.*

The result will be: We shall be able to follow Hahnemann's advice, "Follow my precepts, but follow them accurately." We shall individualize more closely every case that presents itself and requires therapeutic assistance. We shall be more enabled to select the homœopathic remedy and administer it in the smallest dose necessary to overcome disease, as many in the past have done and now are doing.

If the homœopathic physicians of the present will study the materia medica as did the master and his immediate pupils, they will, ere long, gain victory upon victory over the enemy. They will heal the curable sick, gently, speedily, and permanently, thereby step by step verifying the teachings of the master. They will become more and more convinced of the truth, the beautiful action, and universal applicability in non-surgical cases of our law of cure. They will spurn this idea of amalgamating light with darkness, truth with error.

They will not be ashamed of their good mother who gave them life, but be proud to be known as homœopathists. They will not care an iota whether the Regulars (?) retain the old code or adopt a new one. They consult *the unerring law of cure.* It would therefore be madness to ask counsel from men who ridicule and oppose this law.

Such men and such work will secure the full confidence of suffering humanity, add to our ranks the noblest and best educated of the profession, and usher in the day in which Regular Medicine (?), of which Sir John Forbes has said: "Medicine is founded on conjecture and improved by murder," shall have become a thing of the past, and *similia similibus curantur* shall have taken its place.

"We," the followers of the illustrious German, the immortal Samuel Hahnemann, whose honored name ornaments the escutcheon of our Society, in the words of Carroll Dunham, "have received from the generation of pupils and successors of Hahnemann the blazing torch which the Prometheus of our system lighted at the altar of Eternal Truth. Our honor depends on the care with which we cherish it, and the state in which we, in turn, transmit it to those who follow us." And further: "Hahnemann was not made of the stuff that could compromise, for personal ease and prosperity, the charter that God had given him for the benefit of the race. He refused to give up one particle of anything which he deemed essential to the purity and perfectness of his system, and so he has left it to us pure and perfect."

We all have heard these noble words, uttered by one of America's most illustrious sons, a man of pure character, clear and transparent as the diamond; a man of broad culture and great literary attainments, of unswerving fidelity to principle, and untiring zeal in behalf of pure, unalloyed Homœopathy; one who does honor to the proud position accorded to him by common consent, which places him side by side with Samuel Hahnemann and Constantine Hering.

We also have seen some of the progress Homœopathy has made. We have borne our part of the brunt of battle so far. Are we becoming faint hearted in the hour of triumph? Will we yield one inch of the hard won-field? *Never!* Are we making the best possible preparation for new conquests? Shall the standard of our cause be carried victoriously onward? Are we determined to "fight it out on this line, if it takes all summer?" Shall we preserve Homœopathy for posterity, pure and perfect, as we have received it?

Then we must be up and doing. We must be true to ourselves, true to our principles, true to the great therapeutic law of our school. We must preserve our identity while the combat is raging, always rallying around the flag, steadily pressing onward. We must at all times face the enemy, never look back, but follow the chief. It will not be to death, *but to victory.*

I have nothing to say about the best methods of studying the materia medica. I merely desired, in plain words, to call your attention to the fact that the one thing needful above all others, in order to become successful in practice and further the cause of true progress in medicine, is a thorough knowledge of materia medica.

Homœopathy represents the advance guard in medicine. It is a child of Providence, Heaven's choicest blessing for the ills and woes of the human family. It being of Divine origin, it is in its nature *unchangeable and indestructible.* A wonderful future awaits it. If we are faithful, we shall, sooner or later, discover new truths in perfect harmony with those already known. There is no danger that we shall have to unlearn anything. Whatever of the pure drug effects on the healthy human organism is known to us of Acon., Ars., Bell., Bry., Cham., China, Nux vom., Puls., and Sulph., and a host of other tried and trusty friends remains invaluable knowledge forever. As we advance in years we grow in knowledge.

The very opposition we meet increases our confidence, for

Homœopathy, like other truth, is a mighty conqueror, overcoming all opposition; its glory is becoming more and more resplendent as time passes by.

Homœopathy, like prohibition in Iowa, has come to stay. [Continued applause.]

He, therefore, that desires to practice medicine in coming time, will act wisely by preparing himself, through earnest and continuous study of the pathogenesy of drugs, to practice in accord with *the law of similars*, bearing in mind that the physician who has the best knowledge of materia medica is best qualified to heal the sick.

Before closing, I wish to say that if we possess all the knowledge the world can impart, and we have not a thorough knowl- of materia medica, we cannot do the work which is absolutely required that Homœopathy be made the medicine of the world.

*Therefore, know thy Materia Medica.*

